# Supplementary figures and images for: Glyphosate-Induced Abscisic Acid Accumulation Causes Male Sterility in Sea Island Cotton
Source: Plants (Basel). 2023 Feb 27;12(5):1058. doi: 10.3390/plants12051058 (PMC10005681; doi:10.3390/plants12051058)

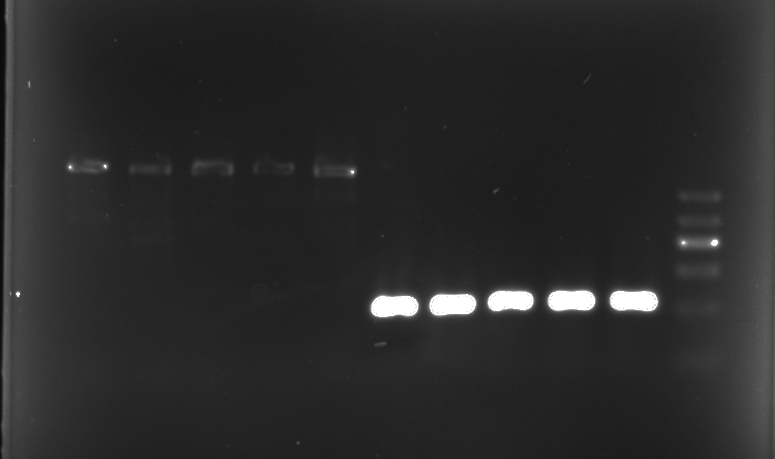

Supplement: Supplementary file 1 [file plants-12-01058-s001.zip › Figure S1.tif]
